# Lowermost Carboniferous (Tournaisian) Miospore Assemblages from the July Field, Gulf of Suez, Egypt: Biostratigraphic and Palaeoenvironmental Implications

**DOI:** 10.3390/life15060872

**Published:** 2025-05-28

**Authors:** Ahmed Maher, Jiří Bek

**Affiliations:** 1 Department of Geology, Faculty of Science, Al-Azhar University, Assiut 71524, Egypt; 2 Laboratory of Palaeobiology and Palaeoecology, Institute of Geology, Academy of Sciences of the Czech Republic, Rozvojová 269, 165 00 Prague, Czech Republic; bek@gli.cas.cz

**Keywords:** Carboniferous, Tournaisian, spores, Gondwana, Gulf of Suez, Egypt

## Abstract

The Nubia Sandstone in the Gulf of Suez, Egypt, is a well-known unclassified sediment. Palynology is considered the most effective tool for dealing with this problem. Miospore assemblages from the Lowermost Carboniferous (Tournaisian) have been discovered from the J62-86 and the J62-64 AST1 wells located in the July Field of the Gulf of Suez, Egypt. Spores are moderately to poorly preserved, suggesting a stratigraphical position within Lowermost Carboniferous ages. The studied sediments include poorly preserved conodont fragments and present significant identification challenges due to the drilling methodologies’ complexities. Spore assemblage consists of 31 genera with 56 species. The dominant spores include zonate genera *Vallatisporites*, *Densosporites*, and *Archaeozonotriletes,* camerate genera *Grandispora*, *Geminospora*, apiculate genera *Apiculiretusispora*, and laevigate trilete genus *Punctatisporites* and megaspores of the *Lagenoisporites* type are recorded. Marine microphytoplankton including *Schizocystia bicornuta*, *Lophosphaeridium*, *Leiosphaerida*, and some filamentous green algae of unknown affinity are recorded. The dispersed spore assemblage is associated with carbonized plant fragments. The palynological data have effectively dated the lower intervals of the Nubia Sandstone from the Nubia “B,” indicating a Lowermost Carboniferous (Tournaisian) age, i.e., *Vallatisporites vallatus–Retustriletes incohatus* palynozone (VI). The stratigraphic differentiation of the Nubia Sandstone is crucial for subsequent correlating subsurface wells in the Gulf of Suez within the context of geology and hydrocarbon exploration, particularly given the scarcity of other fossil groups.

## 1. Introduction

The megaflora and microflora of the Devonian–Carboniferous strata of the eastern part of Egypt have not yet been studied, especially in the eastern part of Egypt in the Gulf of Suez region. This is because of rare Devonian–Carboniferous outcrops and the challenges of finding fossil records in the Nubia Sandstone, representing unclassified sediments from the Cambrian to the Cretaceous age. There were no sufficient fossil records of this complex succession of sediments until today.

The fossil flora, including spores, is crucial for the stratigraphical position of sediments, particularly when fossil fauna and geological indicators are missing. Herein, the palynology is considered the only method for correlating the sedimentary rocks derived from the Nubia Sandstone.

The understanding of the complicated subsurface stratigraphical relations of the Nubia Sandstone remains an argumentative subject despite its significance for the geological framework of North Africa and the Arabian Peninsula. Egypt’s Devonian–Carboniferous stratigraphy is unknown, and no prominent outcrops have been identified. The subsurface well data are the only source of information for investigating this geological system. The evident infrequency of guide fossils within the Palaeozoic clastic sequences in Arabia and Northeastern Africa has significantly challenged their stratigraphic position [1].

### 1.1. The Complexity of the Geologic Setting of the Nubia Sandstone

**Figure 1:**
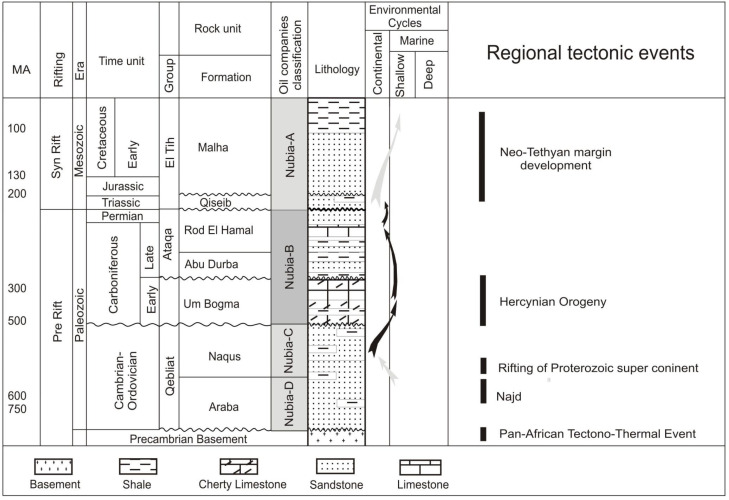
Generalized tectonic and lithostratigraphic column in the Gulf of Suez illustrating the Nubia Sandstone, Egypt (changed from [4,5,6,7,8,9,10,11]). The arrows refer to the environmental cycles.

The Nubia Sandstone (Figure 1) exhibits substantial potential for hydrocarbon exploration. The regional sedimentary sequence of the Nubia Sandstone covers various localities in Egypt and Northeastern Africa, extending throughout Libya, Sudan, Chad, Jordan, Saudi Arabia, and Yemen [2]. Several Palaeozoic outcrops have been documented in the Gulf of Suez region if they relate to the Nubia Sandstone. The Cambrian (Araba Formation) has been described from the Araba–Durba region in Southwestern Sinai and has been identified in the northern sectors of the Abu Gharadig Basin [3].

The Ordovician–Silurian sequences are observed to overlie the Cambro-Ordovician Araba Formation on the western side of the Gulf of Suez [3]. Rare Carboniferous outcrops have been recognized in the Gulf of Suez region. The Early Carboniferous stratigraphic section in the Gulf of Suez Basin has been described from the Um Bogma Formation [12,13]. Although there is no sedimentary record of the Permian formation in Southern Egypt, the Permian might be represented in subsurface well data north of the Western Desert in the Siwa Basin; in the Gulf of Suez Basin, however, the Permian and Triassic formations developed as undifferentiated fluvial sandstone sequences attributed to the Quseib Formation [14]. No palynological data deal with this problem yet.

The Nubia Sandstone “D” member was correlated with the Um Bogma Formation and in the Eastern Sinai. The Nubia Sandstone “C” Member was equivalent to the Abu Thora Formation of the Early to Late Carboniferous in the Um Bogma area, as well as partially correlating with the Rod El Hamal and Abu Darag formations of the Late Carboniferous in Wadi Araba on the western side of the Gulf of Suez. The Nubia “B” member was correlated with the Aheimer Formation of the Late Carboniferous [15]. The Abu Durba Formation of the Early Carboniferous, along with the Aheimer Formation from the Late Carboniferous, exhibits a conformable relationship with the Nubia “B” member; meanwhile, the uppermost section (designated as the Nubia “A” member) correlates with the Permo-Triassic Qiseib Formation located in Wadi Araba, situated on the western flank of the gulf [2,16]. The unconformable surface between the Nubia “C” and the Nubia “B” members shows the non-record of the Silurian and the Devonian ages in the eastern part of Egypt in the Gulf of Suez (Figure 1).

Deficiency in the chronological and stratigraphical dating of the Nubia Sandstone in the Gulf of Suez is known. The objective of this study is to define the undifferentiated sediments obtained from the Nubia Sandstone to gain an understanding of its formation processes and biostratigraphic sections. The study aims to identify the palynomorph assemblages in the Arabian Plate extending into northern Africa, specifically in Egypt.

### 1.2. Previous Work in the Middle East Areas and Egypt

The preceding Palaeozoic palynological studies of the Gulf of Suez and various regions in Egypt have focused on the Carboniferous palynology, as evidenced by the works of [17,18,19,20,21,22,23,24]. Recently, ref. [25] documented significant occurrences of the Carboniferous spore *Vestispora* (Wilson and Hoffmeister) Wilson and Venkatachala in the northwestern desert of Egypt, [26] dated some strata to the Late Emsian to Early Givetian age through the identification of numerous palynomorphs, and [27] investigated spore assemblages believed to belong to the Devonian and Lower Carboniferous from the Kohla-2 well situated in the Western Desert of Egypt. There are insufficient records of the Devonian–Carboniferous boundary in Egypt.

### 1.3. Geologic Setting

**Figure 2:**
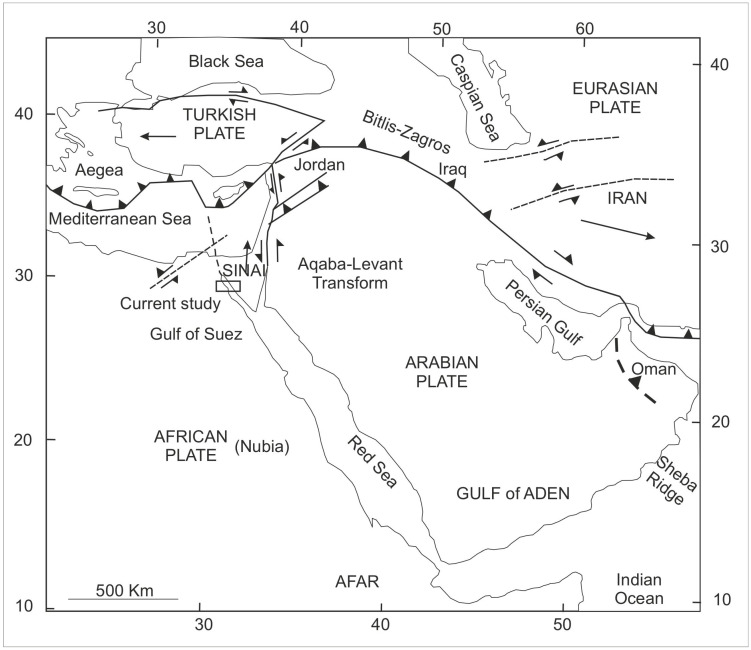
Simplified tectonic setting map showing the location of the Gulf of Suez, Middle East, with an illustration of plate motions (changed from [4,5]).

**Figure 3:**
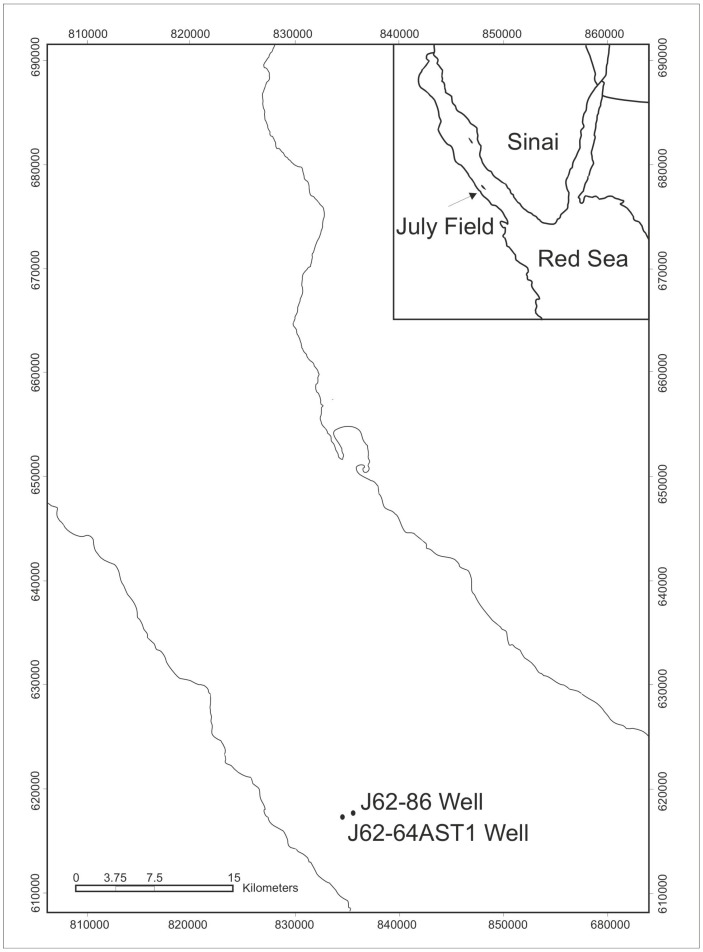
Location map in the July Field showing the J62-86 well and the J62-64 AST1 well [31,32]. The arrow refers to the July Field.

The Gulf of Suez is located between the African and Arabian tectonic plates (Figure 2); it has been studied for oil and gas exploration [28]. The contemporary Gulf of Suez rift, along with the Red Sea oceanic basin and the Aqaba–Dead Sea transform systems, constitutes the Sinai triple junction (Figure 3), which originated during the northeastward displacement of the Arabian plate from the African plate [9]. The Gulf of Suez represents a large graben/half graben assemblage situated between two basement uplifts—the Sinai and the Eastern Desert mountain ranges [10]. The Palaeozoic–Lower Cretaceous Nubian Sandstone consists of a substantial sequence of clastic and thin carbonate sediments, reaching depths of up to 1200 m [11]. The subsurface strata in the Gulf of Suez are recognized as the Nubia Sandstones [11]. The July Field (Figure 3) is regarded as one of the principal oil fields in the Gulf of Suez, Egypt [29]. The Nubia Sandstone exhibits thick stratifications ranging from the Palaeozoic to the Mesozoic and is positioned unconformably on the top of the Precambrian basement complex; this formation is distributed throughout the Gulf of Suez with variable thicknesses, with these strata being exposed along the western and eastern margins of the Gulf of Suez [9]. The characteristics of the Nubia Sandstone are consistent and situated in proximity to the northern boundary of the African craton [30].

### 1.4. Stratigraphy of the Pre-Rift

**Figure 4:**
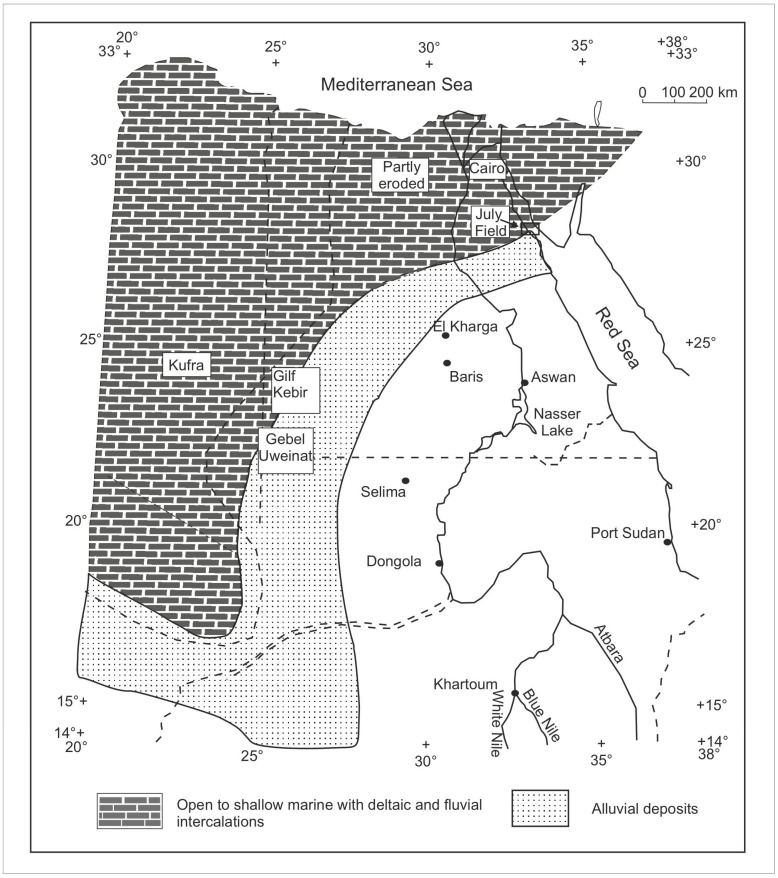
Distribution and facies of Lower Carboniferous sediments in northeast Africa and approximate extension of marine influence in Devonian time, in addition to the study of the July Field [30]. The arrow refers to the July Field.

The pre-rift stratigraphic sequences in the southern Gulf of Suez are characterized by a basal sandstone layer, which is subsequently overlain by an assemblage of interbedded shale, sandstone, and limestone (Figure 2 and Figure 4). The basal section of the Nubia, in certain locations, is constituted of several tens of meters of interbedded red siltstone and sandstone that remains undated. The Palaeozoic succession of the Nubia Sandstone encompasses a series of siliciclastic and sedimentary intervals whose depositional characteristics are indicative of shallow marine to fluvio-deltaic environments [10,11].

The Palaeozoic rocks exposed in the Um Bogma area in the Southwestern Sinai have been discussed in [23]. The correlation between the surface and subsurface Palaeozoic complex is illustrated in (Figure 1). The Ataqa Group (Figure 1) was used to describe different sections in Central Sinai boreholes and in the Eastern Desert [15]. The Ataqa Group is underlain by the Qebliat Group (Figure 1), and these units are dominantly clastic and were proposed to belong to the Cambro-Ordovician based on their trace fossil contents [23]. The interval between the Cambrian and Carboniferous strata (Figure 1) is poorly dated, and this unit was named Nubia “C” [15]. This unit of Nubia “C” is well represented in the July Field of the current study, and it was proposed to be of the Ordovician, Silurian, and Devonian age [15]. In Southwestern Egypt and Northwestern Sudan, the Devonian is not dated palaeontologically. However, part of the strata overlying the Silurian beds at Gebel Tageru, east and southeast of Gebel Kissu, and at Gebel Uweinat (Figure 4) were proposed to be of the Devonian age [30], while in northwestern Egypt, there were some palynomorphs reported to be of Late Emsian to Early Givetian age [26].

## 2. Materials and Methods

**Figure 5:**
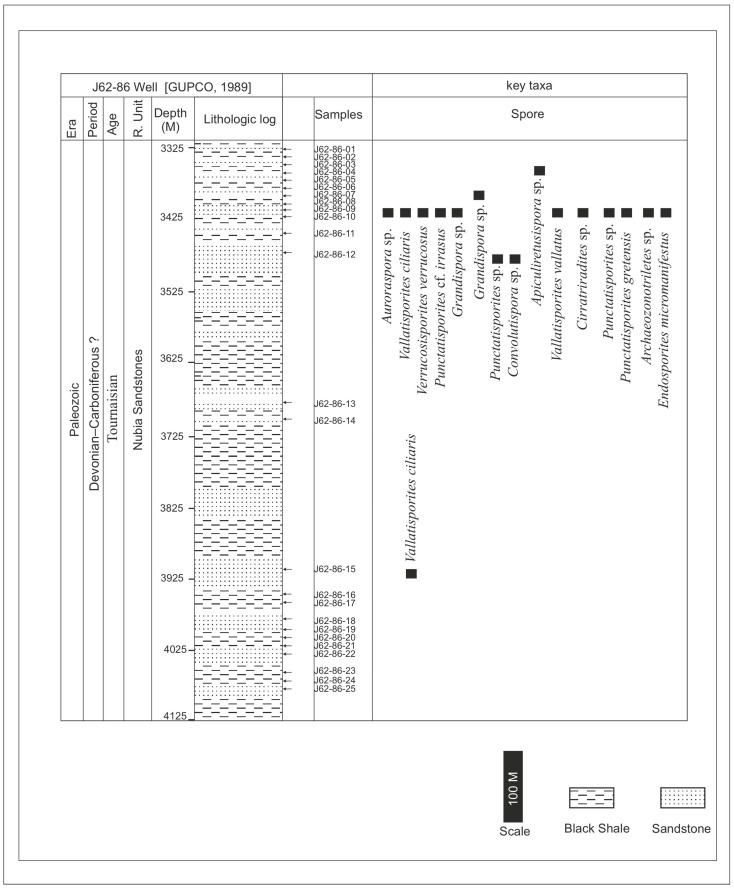
Lithologic log and chronostratigraphic framework of the J62-86 well, Gulf of Suez, Egypt (log based on [31]), with key taxa. The arrows refer to the location of the studied samples.

**Figure 6:**
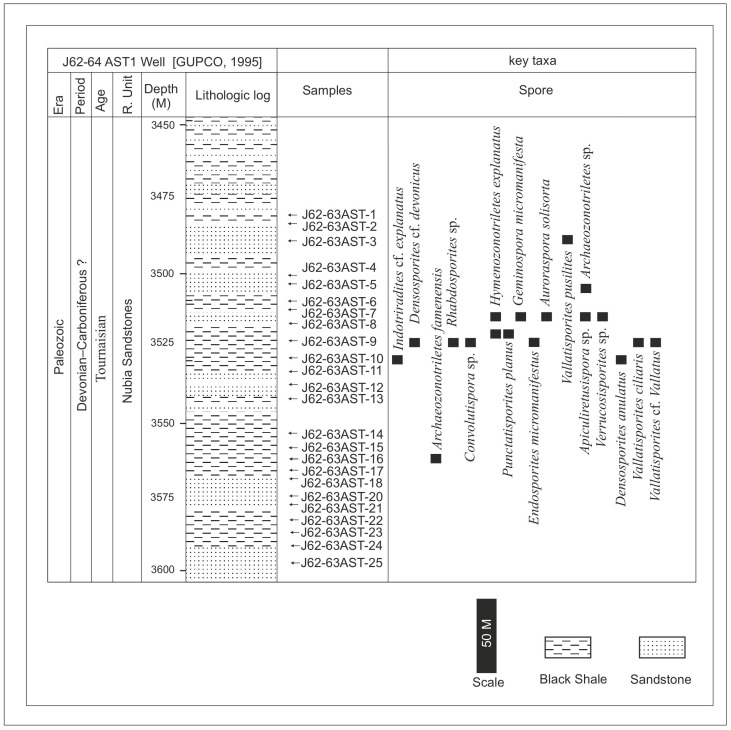
Lithologic log and chronostratigraphic framework of the J62-64 AST1 well, Gulf of Suez, Egypt (log based on [32]), with key taxa. The arrows refer to the location of the studied samples.

The current samples were chosen from minor shale interlayers embedded in extensive sandstone sequences [10,11], and the location of the samples is illustrated (Figure 5 and Figure 6). A total of fifty cuts of ditch samples were collected; no core samples could be obtained from the Gulf of Suez Petroleum Company (GUPCO). The samples are ditch-cutting samples, so the risk of caving is probable. These comprise shaly sand-sourced samples, which might be from the Nubia C and the Nubia B members. The geographic position of the J62-64 AST1 well is latitude 28.14′24.54″ N and longitude 33.16′46.32″. The geographic position of the J62-86 well is latitude 37′1.34″ N and longitude 50′19.42″.

The ditch samples were initially described and studied using hand specimens and a binocular optical microscope. The lithologic analysis performed under a light microscope reveals that the obtained samples are predominantly composed of very fine sediments composed of dark black shale and sandstone. The samples were prepared for the palaeopalynological analysis according to the standard tools [33]. The obtained samples noticed that it was affected by the oil, not knowing if it was from the reservoir intervals. The samples were washed with acetone before acid treatments. The sample treatment (5 g) involved HCI-HF-HCL processing and sieving through 10 μm meshes. Some samples were also macerated in nitric acid, but the preservation has still not improved. Slide preparation included the use of ICL’s Elvacite^®^ 4345: DuPont America, Wilmington, DE, USA, and some slides used glycerin jelly.

The July Field samples were prepared for conodonts. Each sample was washed with water and put on a hot plate for two hours, then left to be returned to the lab at the natural temperature. Each sample sieve was investigated under a light Optika microscope. Conodont elements were observed and isolated for SEM. Most isolated samples were missing the main taxonomic features for the identification process, which might be because of the drilling process. An isolated conodont element was tested with HCL; however, no effect was detected. Microfossils designated for SEM examination were affixed on aluminium stumps, subsequently coated with platinum via a magnetron sputter coater (JUC-5000, JEOL, Tokyo, Japan), and scrutinized under an SEM (JSMIT100, JEOL). JEOL Ltd., Akishima, Tokyo, JEOL. These SEM observations were conducted at Japan’s Shizuoka Museum for Natural and Environmental History. Residual materials, slides, and original cutting samples are securely housed at the Department of Geology, Al-Azhar University (Assiut), Egypt. A list of recorded taxa is provided in Appendix A. The present manuscript is attached with the Supplementary File.

## 3. Results

**Figure 7:**
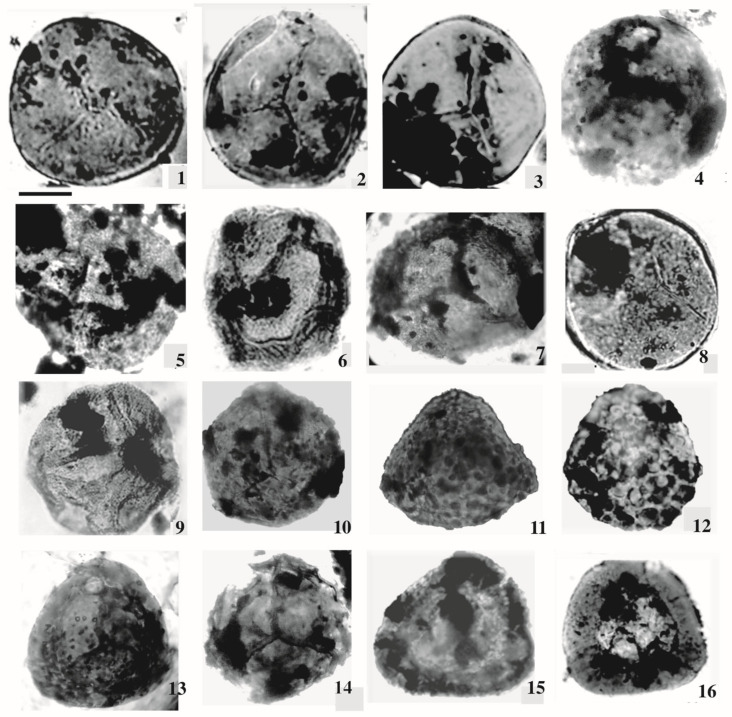
1. *Punctatisporites gretensis* Balme and Henelly; J62-64 AST1 well. 2. *Punctatisporites* sp.; J62-64 AST1-012-D2: EF. V26/2. 3. *Punctatisporites planus* Hacquebard; J62-86-09-D2: EF. C42/3. 4. *Punctatisporites* sp. 5. *Verrucosisporites* sp.; J62-64 AST1-08F1. 6. *Verrucosisporites* sp.; J2-07-1. 7. *Apiculiretusispora arabiensis* Al-Ghazi. 8. *Punctatisporites* cf. *irrasus* Hacquebard; J62-86-010-1. 9. *Punctatisporites* sp.; J2-03-1. 10. *Retusotriletes* cf. *incohatus* Sullivan. 11. *Pustulatisporites* sp. 12. *Verrucosisporites verrucosus* (Ibrahim) Ibrahim; J62-86-09-AZ-16. 13. *Microreticulatisporites* sp. 14. *Retusotriletes warringtonii* Richardson and Lister. 15. *Densosporites* cf. *devonicus* Richardson; J62-86-10-1. 16. *Densosporites anulatus* (Loose) Schopf, Wilson and Bentall; J62-64 AST1-010-D1: EF. D40. Scale bar = 10 μm. The samples in our study were obtained from the deepest wells in the Gulf of Suez, which were significantly influenced by the reservoir rock, leading to poor preservation despite multiple extraction attempts. To improve quality, we remacerated some samples and captured new photomicrographs, which were incorporated into the manuscript.

**Figure 8:**
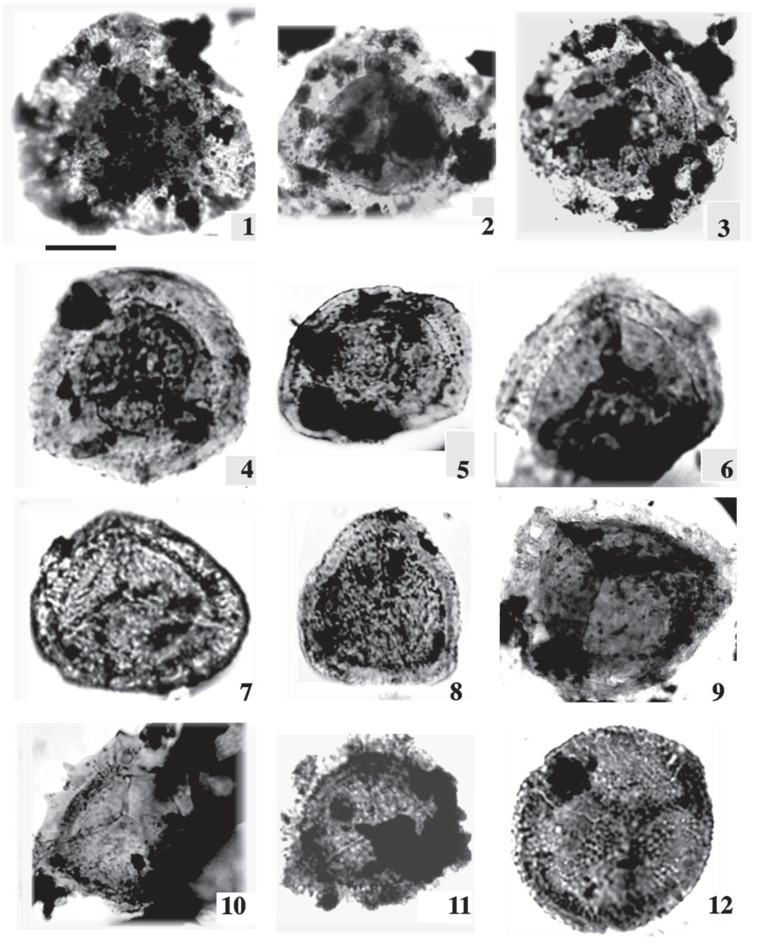
1. *Auroraspora solisorta* Hoffmeister, Staplin and Malloy; J62-64 AST1-08-6 and J1-08-A1: EF. C20/2. 2. *Indotriradites* cf. *explanatus* (Luber) Playford; J62-64 AST1-010-A: EF. F51. 3. *Auroraspora* sp.; J62-86-010-1. EF. F51. 4. *Rhabdosporites* sp.: J62-86-09-B-6: P39/4 and J2-020-L1: EF. X1. 5. *Vallatisporites* sp.; J62-86-09-D1-1. 6. *Vallatisporites* sp.; J62-86-09-B-6: EF. E34. 7. *Geminospora* cf. *lemurata*; J62-86-09-E-2: EF. J51/4. 8. *Geminospora* sp.; J62-86-09-5: EF. C60-4. 9. *Vallatisporites* cf. *pusillites*. 10. *Ancyrospora* sp.; J62-64 AST1-012. 11. *Cirratriradites* sp.; J62-86-09-A-9: EF. B36. 12. *Archaeozonotriletes* cf. *famenensis* Naumova; J62-86-016-1. Scale bar = 10 μm. The samples in our study were obtained from the deepest wells in the Gulf of Suez, which were significantly influenced by the reservoir rock, leading to poor preservation despite multiple extraction attempts. To improve quality, we remacerated some samples and captured new photomicrographs, which were incorporated into the manuscript.

**Figure 9:**
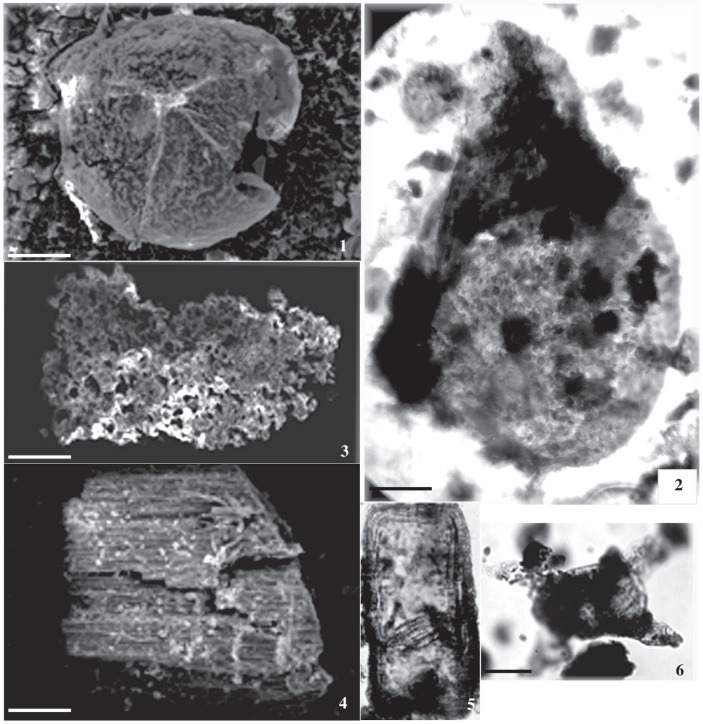
1. Trilete unidentified megaspore, SEM, scale bar = 100 μm. 2. Trilete megaspore of the *Lagenoisporites* type. Note the prominent apical gula (upper structure), SEM, scale bar = 100 μm. 3. Cuticle-like sheet or pseudocellular cuticle, SEM, scale bar = 100 μm. 4. Wood fragment, SEM, scale bar = 100 μm. 5. Filamentous green algae; J2-05-1: EF. G9, scale bar = 10 μm. 6. *Schizocystia bicornuta* Jardinè; J62-86 well, scale bar = 10 μm. The samples in our study were obtained from the deepest wells in the Gulf of Suez, which were significantly influenced by the reservoir rock, leading to poor preservation despite multiple extraction attempts. To improve quality, we remacerated some samples and captured new photomicrographs, which were incorporated into the manuscript.

**Figure 10:**
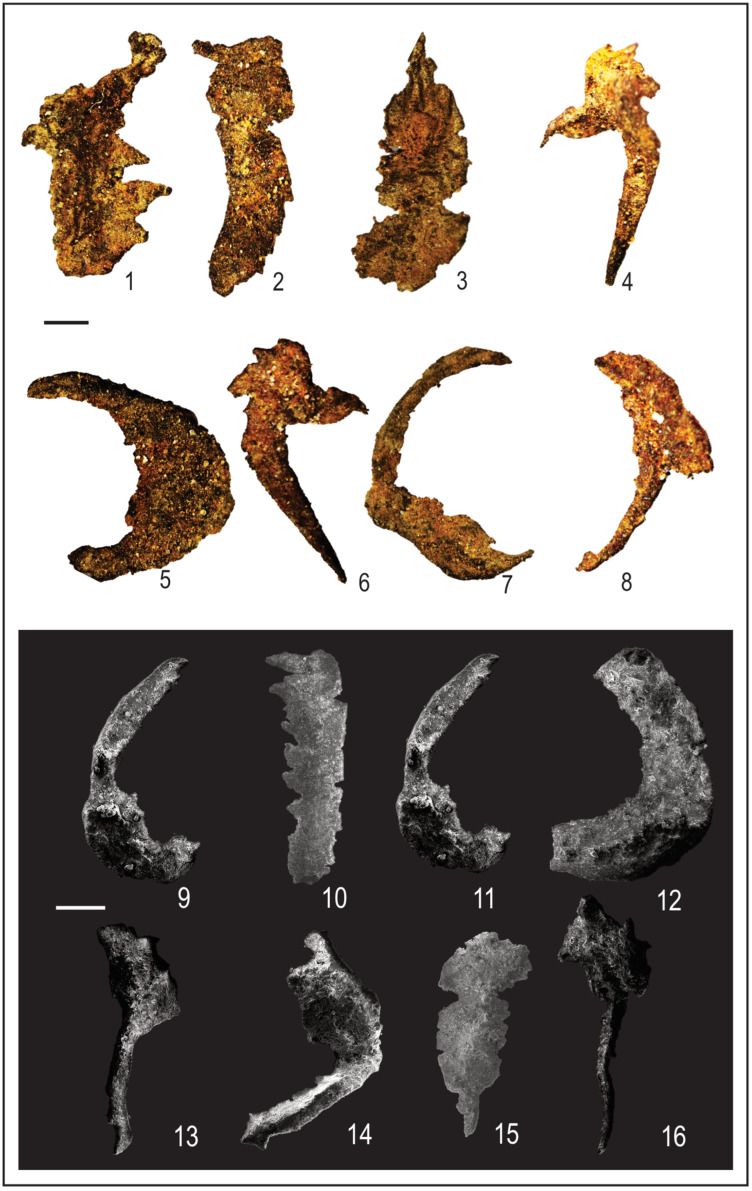
The drilling process caused unidentified conodont assemblage from 1 to 7. Photomicrographs of conodont fragments: 8–16. Conodont fragments: 1–7 (Scale bar 400 μm).

The current investigation of the dispersed spore assemblage has studied the spore assemblage of the Nubia Sandstone deposits in the J62-86 well and the J62-64 AST1 wells (Figure 3 and Figure 4) to assign a palynostratigraphic framework for the Middle Palaeozoic succession. The spore succession in the studied wells is based on ditch cutting with the probability of downhole contamination. The results obtained have partially been supported by conodont microfossils but without identification because the logging tools destroyed most of the taxonomic features. The palynological and conodont assemblages are illustrated and represented in Figure 5, Figure 6, Figure 7, Figure 8, Figure 9 and Figure 10.

The studied samples from the J62-64 AST1 well and J62-86 well exhibit a diverse range of sporomorphs, with very few palynomorphs. The palynological investigation of the July Field reveals that the preservation of spore assemblages is moderate to poor (dark brown), with productive samples being concentrated in specific intervals (Figure 5 and Figure 6). Out of fifty processed samples from the July Field in the Gulf of Suez, only a few were productive and yielded spores. The accurate stratigraphic setting of the studied samples from the July Fields will rely on the spore assemblage. The poor preservation of conodont elements prevents firm identification, but they are presumed to be of the same age as the recovered spore assemblage.

Dispersed spore assemblages occur only in some restricted intervals and not in the whole profile (Figure 3 and Figure 4). The most productive samples in J62-64 AST1 well were at depths [3500, 3525, and 3530 m] of samples [8,10,12]. The productive interval in J62-86 well is [3425, and 3450] of samples [9,10]. Zonate and camerate spores, microgranulate and verrucate spores of the genus *Verrucosisporites* (Ibrahim) Smith and Butterworth, and spores of the genus *Hymenozonotriletes* Naumova dominated. Moreover, some megaspores were observed in the genus. Abundant are plant fragments, tracheids, pseudocellular cuticles, cuticles of arthropods, and tubular fragments. Sculptured spores of the genus *Apiculiretusispora* (Streel) Streel laevigate and patinate *Archaeozonotriletes* (Naumova) Allen, crassitate forms including *Aneurospora* (Streel) Richardson et al., and ornamented patinate spores (e.g., *Cymbosporites* Allen) are rare. The spore assemblage is characterized mainly by spores of genera *Apiculatisporites* (Ibrahim) Potonié and Kremp, *Convolutispora* Hoffmeister et al., *Densosporites* (Berry) Butterworth et al., *Geminospora* (Balme) Playford, *Grandispora* Hoffmeister, Staplin and Malloy, *Punctatisporites* Ibrahim, *Pustulatisporites* Potonié and Kremp *Rhabdosporites* Richardson, *Samarisporites* Richardson, *Spelaeotriletes* Neves and Owens, *Vallatisporites* (Hacquebard) Sullivan*, Chelinospora* Allen*,* and *Synorisporites* Richardson and Lister, some *Reticulatisporites* Ibrahim, and several representatives of *Leiosphaerida* Eisenack, *Lophosphaeridium* Timofeev, *Schizocystia bicornuta* Jardiné, and fragments of filamentous green algae.

The palynofacies evidence suggests that the spore assemblage was deposited near marine settings, as indicated by conodonts. Additionally, the dominance of plant remains provides further evidence of the near source of the vegetational cover.

## 4. Discussion

### 4.1. The Stratigraphical Position of Spore Assemblage in the Gulf of Suez, Egypt

The July Field spore assemblage contains marker spore taxa of the Devonian and the Carboniferous ages. The unconformity surface between the Nubia “C” and Nubia “B” in (Figure 1) is interesting because it could bear new information for the missing ages of the Devonian or the Devonian–Carboniferous boundary.

The Palaeozoic marine transgressions that occurred during the Early to Middle Palaeozoic accelerated the reworking and extensive dispersal of siliciclastic sediments [3]. The distribution of the Lower Carboniferous sediments in Northeast Africa and the extension of marine influence in the Devonian time is illustrated in (Figure 4), which declares that the study of the July Field area was in an open, too shallow marine environment with deltaic and fluvial interactions [30].

The July Field spore assemblage suggests being younger than the Uppermost Devonian age. This is because [34,35] concur that stratigraphically important *Retispora lepidophyta* (Kedo) Playford is classified to the Uppermost Devonian, and it is not documented in the July Field spore assemblage. However, the appearance of *Verrucosisporites nitidus* (Naumova) Playford defines the base of the Uppermost Devonian *Retispora lepidophyta*–*Verrucosisporites nitidus* (LN) Biozone in Western Europe, with *Vallatisporites verrucosus* (Ibrahim) Ibrahim typically evolving at the same stratigraphic level [35]. *Verrucosisporites nitidus* has been regarded as the most recent Devonian characteristic microspore associated with *Retispora lepidophyta* [36]. Spore assemblage exhibits similarities to that noted from the latest Devonian and Early Carboniferous microspore assemblage in Saudi Arabia [35]. The assemblage zone characterized by *Verrucosisporites nitidus* and *Vallatisporites verrucosus*, as documented by [37], is situated within the (Late Strunian) Late Famennian age. This specific zone is distinguished by the prevalence of characteristic species that exhibit occurrences in combination with the taxa currently recorded from the July Field. The species of note within this assemblage include *Verrucosisporites nitidus*, *Vallatisporites verrucosus*, and *Indotriradites* cf. *explanatus* (Luber) Playford.

The microspores obtained from the July Field can be correlated with the *Vallatisporites pusillites–Retusotriletes incohatus* assemblage zone [37]. This zone has been dated to encompass the Tournaisian age. The current spore assemblage from the July Field shares the occurrence of genera, including *Auroraspora* Hoffmeister et al.; *Archaeozonotriletesi* cf. *famenensis* Naumova, *Grandispora*, *Archaeozonotriletes*; *Retusotriletes* cf. *incohatus*; and *Hymenozonotriletes.* however, the associated taxa between the [37] and the July Field wells in the current study recommend the Tournaisian age.

The July Field assemblage is characterized by the appearance of the monopseudosaccate spore without foveoreticulate exoexine, such as *Auroraspora*, and *Grandispora*, which is considered the description of the *Rugospora flexuosa*–*Grandispora cornuta* assemblage zone [37]; this represents partial correlation.

The Givetian–Frasnian boundary deposits in Byelarus were proposed to be distinguished by a marked palynofloral shift [38,39]. *Verrucosisporites nitidus* is recognized as a marker species indicative of the Devonian–Carboniferous boundary [40,41]. The record of this species in the current spore assemblage in the July Field suggests that the July Field could be located between the Nubia “C” and the Nubia “B. This refers to the probability of the Devonian–Carboniferous transition interval that occurred in the July Field.

Ref. [42] investigated the Carboniferous assemblages from the Kingdom of Saudi Arabia and identified them as Tournaisian, mainly characterized by *Indotriradites* cf. *explanatus*, *Auroraspora macra* Sullivan, *Punctatisporites irrasus* Hacquebard, and various other taxa, including *Calamospora* Schopf et al., *Knoxisporites literatus* (Waltz) Playford, *Perotrilites perinatus* Hughes and Playford, *Retusotriletes* (Naumova) Streel, *Vallatisporites vallatus* Hacquebard, and *V*. *verrucosus*. These spores are also recorded in the studied samples from the July Field, with the absence of the uppermost Devonian taxon *Retispora lepidophyta* being unobserved both in the July Field and the assemblage documented by [38]. Furthermore, the Early Carboniferous spore and pollen taxa noted from AR’AR-1 well by [42] were not represented in the July Field study.

The Gondwana Devonian and Carboniferous palynological records reveal crucial insights into its paleobiogeographical and palaeoclimatic evolution [43,44,45].

The assemblage zone A, recorded from the Carboniferous strata of the Abu Rodeiyim boreholes [24] in West-Central Sinai, Egypt, assumes significant resemblance to the spore assemblage currently documented from the samples analysed in the July Field. There are shared occurrences of *Punctatisporites*, *Verrucosisporites*, *Convolutispora*, *Vallatisporites*, and *Densosporites*, and this assemblage has been assigned to the Late Viséan age, primarily in regions encompassing North Africa, Western Europe, and Western Australia.

The observation of the megaspore in the July Field miospore assemblage is considered to represent an indication that it might indicate the presence of arborescent bisporangiate lycophytes [46,47] in the Gulf of Suez, Egypt. Trilete gulate megaspores of the *Lagenoisporites* type are recorded among other megaspores.

The record of *Schizocystia bicornuta* in the July Field wells was recorded from the Upper Devonian of Algeria as a guide fossil [48]. Filamentous green algae observed in the July Field studied samples have great similarity to the individual specimens morphotypes reported from the Early Devonian Rhynie chert, which inhabited freshwater ponds/lakes [49]. This assumption can greatly agree with the new reconstructed palaeodepositional environment for the July Field inferred from [30], which indicates that the area was of the deltaic, fluvial, and alluvial origin. The presence of cuticle-like sheets, cuticles of arthropods, plant remains, and tracheids are consistent with this assumption. The occurrences of genera *Lophosphaeridium* and *Leiosphaerida* are indicated in the shallow water environment. The July Field miospore assemblage could be assumed to have been transported from the vegetation source by the deltaic system.

#### Comparison with Other Dispersed Spore Assemblages

**Figure 11:**
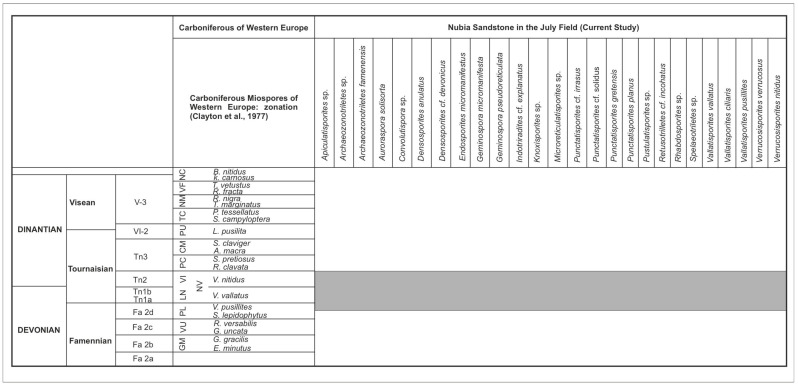
Correlation of the recorded spore assemblage with the zone of the Old Red Sandstone continent and adjacent regions, modified from [59]. The grey box refers to the correlated zone of the Carboniferous of Western Europe with the July Field in the Gulf of Suez (Current study), Egypt.

The recorded spore assemblage from the currently studied July well samples is correlated with the areas from the Gondwana and the Old Red Sandstone continent and adjacent regions [37]. The next section will discuss the correlation, and the established zonation is constructed in (Figure 11). The current spore assemblage is correlated with the Old Red Sandstone continent [37].

The documented spore assemblages from the Western Argentine and Southern Bolivian regions, dating to the Middle Devonian age, are indicative of the established patterns within the Afro-South American Subrealm [37]. The identification of *Grandispora pseudoreticulata* in the present investigation of the July Field from the Gulf of Suez, Egypt, serves to corroborate the hypothesis that the extended patterns of the Afro-South American Subrealm encompass Egypt within these palaeogeographical contexts of Gondwana [43]. This taxon, *Grandispora pseudoreticulata*, has also been reported from the Middle Devonian strata of Northwestern Argentina [38]. Furthermore, it has been documented within the Mid-Late Devonian assemblages of herbaceous lycophytes from Northern Argentina and Bolivia [43]; however, the infrequent presence of some taxa does not help to establish a correlation [44]. This indicates that the biogeographical patterns of the Afro-South American Subrealm could extend to include or reach Egypt within these palaeogeographical regions of Gondwana [44].

The comparative analysis with the palynostratigraphic studies conducted by [37] is related to the present spore assemblage acquired from the July Field in the Gulf of Suez, Egypt. The *Vallatisporites pusillites sensu lato* and *Retispora lepidophyta* zone, as characterized by [37], have been attributed to a Late Famennian (Strunian) age. This assemblage is characterized by the presence of *Vallatisporites pusillites*, and dominance of *Vallatisporites* demonstrates notable similarities to the findings presented in the current study of the July Field. The *Vallatisporites pusillites* encountered in the present study are well documented across numerous locations, including various regions in North America, such as Western New York State, Pennsylvania, and international locations, including Belgium, Kazakhstan, Tibet, South China, the former U.S.S.R., Eastern Europe, and Western Europe [37].

*Indotriradites* cf. *explanatus*, previously recorded from locations in Ireland, Southern Ireland, the Ardennes–Rhine region, Western New York State, and Northern Pennsylvania [37], has also been identified in the current study conducted at the July Field.

In addition to another zone delineated by [37], the *Verrucosisporites nitidus–Vallatisporites verrucosus* assemblage zone corresponds to the Famennian (Strunian) age. This assemblage is distinguished by the presence of *Verrucosisporites nitidus*, *Vallatisporites verrucosus*. A comparable occurrence of *Verrucosisporites riitidus*, *Vallatisporites verrucosus*, *Discernisporites*, *Grandispora*, *Indotriradites* cf. *explanatus*, and *Knoxisporites* has been documented in the present study conducted in the July Field.

The abundance of the *Vallatisporites* in the present study can highly recommend the transition between the Devonian and Carboniferous periods. However, several European localities noted the *lepidophyta* group’s extinction [45]. The non-recorded *lepidophyta* group in the July Field could be caused by some changes in the palaeoenvironment of the deposits. The disappearances of the *Retispora lepidophyta* were proposed to be of the Devonian–Carboniferous characteristic boundary [50]. We could not confirm that similar conditions controlled the Hangenberg Shales [45] if extended to the July Field. However, the infrequent occurrences of marine micophytoplankton in the July Field could be questionable.

*Apiculiretusispora plicata* (Allen) Streel was recorded from the palynological assemblage of the Lower Devonian of China [51,52]. *Geminospora libyensis* Richardson was recorded from the Devonian spores of South China, Saudi Arabia, and Australia [53]. The occurrence of megaspores of the *Lagenoisporites* type is important for the spore evolution in the Devonian–Carboniferous of Egypt and means the occurrence of first arborescent lycophytes, i.e., bisporangiate members of the *Lepidophloios* family [54] in Egypt. The megaspore was proposed to be regarded as the event that occurred to coincide with the first occurrence of the distinctive group of Famennian species of *Diducites* [55]. The affinity of this important megaspore is attributed to Lepidodendraceae [56]. *Apiculiretusispora arabiensis* Al-Ghazi was recorded from the Emsian of Saudi Arabia [57] and from Lower Devonian Rañeces—La Vid groups of Northern Spain [58].

The location of the study of the July Field (Figure 3 and Figure 4) in the distribution facies of the Lower Carboniferous sediments in Northeast Africa and the extension of the marine influence in the Devonian age [30] illustrating the palaeodepositional setting of the study area of the July Field are pictured. The July Field palynological assemblage contains spores, rare marine microphytoplankton, plant fragments, and cuticle sheets. In addition to the presence of the conodont elements. This July Field assemblage agrees with the facies of the deltaic and fluvial intercalations of the Devonian age extended in North Africa, including the Gulf of Suez, Egypt. The spore assemblage is greatly represented by the dominance of the early lycophytes and some bryophytes or early tracheophytes, with the suggestion of the emergence of the early ferns.

### 4.2. The Tournaisian Spore Assemblage in the Gulf of Suez, Egypt

The correlation of the present spore assemblage obtained from the July Field in the Gulf of Suez can be correlated to the *Vallatisporites vallatus*–*Retusotriletes incohatus* (VI) Subzone [59]. The present July spore assemblage is correlated with the palynological Western Europe zonation [59]. The *Vallatisporites pusillites*–*Spelaeotriletes lepidophytus* (PL) Zone is characterized by the presence of *Spelaeotriletes lepidophytus* (Kedo) Streel and *Vallatisporites pusillites* (Kedo) Dolby and Neves, while *Spelaeotriletes lepidophytus* is very abundant [59]. These spore taxa are recorded from the present study in the July Field wells. Another significant feature that the spore assemblage recorded in the (PL) zone of [59] is containing poorly preserved spores of the Devonian, while the well-preserved spores are thought to belong to the Carboniferous in Tournaisian age [59], this feature is also noticed in the July Field. The Devonian–Carboniferous boundary was thought to be in the (PL) zone, sharing the same idea for the present July spore assemblage.

There is a correlation with the *Verrucosisporites nitidus*–*Vallatisporites vallatus* (NV) zone [59] with the present July spore assemblage containing *Indotriradites* cf. *explanatus* with *Spelaeotriletes lepidophytus* (Kedo) Streel, *Vallatisporites pusillites*, and *Rugospora flexuosa*. The (NV) zone [59] shares the same taxa with the July spore assemblage.

There is a correlation with the *Spelaeotriletes lepidophytus*–*Verrucosisporites nitidus* (LN) subzone containing *Spelaeotriletes lepidophytus*, *Retusotriletes incohatus* Sullivan, *Verrucosisporites nitidus*, *Raistrickia macrura* (Luber) Dolby and Neves, *Raistrickia variabilis* Dolby and Neves, *Dictyotriletes submarginatus* Playford, *Hymenozonotriletes commutatus* Naumova, *Lophozonotriletes malevkensis* Kedo, *Vallatisporites pusillites*, *Vallatisporites verrucosus*, *Auroraspora macra* Sullivan, *Auroraspora poljessica* (Kedo) Streel, *Discernisporites micromanifestus* (Hacquebard) Sabry and Neves, and *Rugospora flexuosa*. The (LN) subzone of [59] is sharing the findings of the main spore taxa in the (LN) subzone except for the non-record of *Raistrickia macra*, *Raistrickia variabilis*, *Dictyotriletes submarginatus*, *Hymenozonotriletes commutatus*, *Lophozonotriletes malevkensis*, *Vallatisporites pusillites*, *Vallatisporites verrucosus*, *Auroraspora macra*, *Auroraspora poljessica*, *Disernisporites micromanifestus*, and *Rugospora flexuosa*.

The base of the VI subzone is defined from the Munster Basin, Germany, the Kinsale Formation, and it represents the upper part of the “Old Red Sandstone” and the lower part of the Porter’s Gate Formation at Hook Head, Co. Wexford, and the lower part of the Lower Limestone Shales in the Mendips, S.W. Britain, UK [59]. This subzone is defined by the disappearances of *Spelaeotriletes lepidophytus* Owens and Streel together with *Vallatisporites pusillites* Hacquebard and *Rugospora flexuosa* Higgs [59]. The lowermost assemblage of this subzone is dominated by smooth acamerate spores *Punctatisporites irrasus* Hacquebard and *Retusotriletes incohatus* Hacquebard, *Lophozonotriletes cristifer* (Luber) Kedo, *Retusotriletes triangulatus* (Streel) Streel, *Pulvinispora quasilabrata* Higgs, *Raistrickia corynoges* Sullivan (Naumova) Playford, *Raistrickia corynoges* Sullivan, *Convolutispora* sp., *Corbulispora cancellata*, *Dictyotrilletes submarginatus*, *Indotriradites* cf. *explanatus*, *Knoxisporites literatus* (Waltz) Playford, *Lophozonotriletes cristifer* (Luber) Kedo, *Lophozonotriletes malevkensis* Birina, *Lophozonotriletes rarituberculatus* (Luber) Kedo, and *Latosporites* sp. *Dibolisporites distinctus* (Clayton) Playford appears but is not common. *Spelaeotriletes balteatus* (Playford) Higgs is frequently abundant. [59]. The listed taxa of the (VI) subzone of [59] are recorded in the present spore assemblage in the July Field in the Gulf of Suez, Egypt, except some taxa are not observed. These are *Spelaeotriletes lepidophytus*, *Rugospora flexuosa*, *Lophozonotriletes cristifer*, *Pulvinispora quasilabrata*, *Raistrickia corynoges*, *Corbulispora cancellata*, *Dictyotrilletes submarginatus*, *Lophozonotriletes cristifer*, *L.s malevkensis*, *L. rarituberculatus*, *Dibolisporites distinctus*, and *Spelaeotriletes balteatus*.

The correlation with the Carboniferous spore zonation [59] shares many similarities to the present July spore assemblage: these zones are the *Vallatisporites pusillites*–*Spelaeotriletes lepidophytus* (PL) zone, *Verrucosisporites nitidus*–*Vallatisporites vallatus* (NV) zone, *Spelaeotriletes lepidophytus–Verrucosisporites nitidus* (LN) subzone, and VI subzone of the *Vallatisporites vallatus*–*Retusotriletes incohatus*. The July study well spore assemblage is well established to be correlated to the PL zone, NV zone, LN subzone, VI subzone [59]. This confirms that the July study wells are of the Tournaisian age.

**Figure 12:**
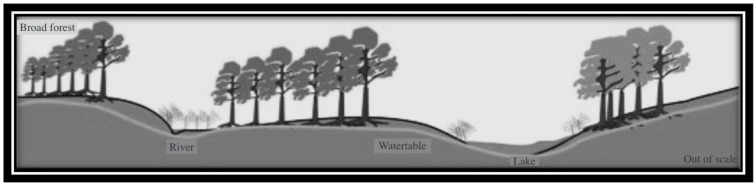
A simple reconstruction of the palaeovegetation and palaeoenvironment of the Nubia sandstone from the study of the July Field in the Gulf of Suez, Egypt.

The present July spore assemblage is older than the study of the regions of Sinai and the Gulf of Suez [17,19,20,21,22,23,24], which were reported to be of the Visean to Late Carboniferous age. The presence of megaspores in the July Field spore assemblage indicates a significant evolutionary event in the palaeovegetation of Egypt. It could refer to the evolution of the seed plants, of which it could mark the diversity of plant proliferation of various groups. Reconstruction of the palaeovegetation of the Lowermost Carboniferous Nubia sandstone in the Gulf of Suez, Egypt, is introduced (Figure 12).

The correlation within the upper Devonian and Carboniferous miospore assemblage suggests that the July Field spore assemblage is more suitable to be compatible with the Lowermost Carboniferous age. The stratigraphic setting of the Nubia sandstone is complex and not resolved before. The location of the Nubia “B” in the lithostratigraphic column in the Gulf of Suez (Figure 1) illustrating an unconformity surface for the lower interval of the Nubia “B” [4,5,6,7,8,9,10,11]. The observation of the Devonian and Carboniferous spore taxa in the lower interval of the study wells from the July Field indicates the Devonian age, but the July Field is palynologically more consistent to the Lowermost Carboniferous than the uppermost Devonian ages. The July Field study wells are palynologically dated to the Tournaisian age.

## 5. Conclusions

For the first time, the palynological investigations have enabled the description of lithostratigraphic and palynostratigraphic units within the subsurface layers of the Nubia Sandstone. The established palynostratigraphic units have been systematically correlated and temporally assigned to the Lowermost Carboniferous (Tournaisian) and could reach the transition interval between the Devonian and Carboniferous boundary. The age dating of the lower interval of the Nubia Sandstone B member, inferred from the correlation of spore assemblages, is assumed to correspond to the Devonian–Carboniferous intervals. Correlation with the (VI) subzone from S.W. Britain can mark the introductory identification of such a stratigraphic classification within the Gulf of Suez, Egypt. The chronological assessment of the analysed samples from the Nubia Sandstone is characterised as the basal interval of the Nubia B member. The stratigraphical position of spore assemblage in the Gulf of Suez, Egypt, is suggested to be located between the lower interval of the Nubia B and the upper interval of the Nubia C.

The analysed specimens have been predominantly characterized by conodont elements, yet taxonomic identification was hindered due to the presumed destructive impact of drilling tools on the morphological features of the collected specimens. The palynological and palaeontological data obtained from the July Field indicate the presence of the deposition of the Devonian–Carboniferous intervals in the Gulf of Suez, Egypt. It is suggested to be of the deltaic, fluvial, or alluvial origin. This conclusion is very important for the understanding of the paleogeography and palaeodeposition of Egypt during the Devonian age.

## Data Availability

Palynological data are available in the database stored at the Department of Geology, Faculty of Science, Al-Azhar University (Assiut), Egypt.

## Supplementary Materials

The following supporting information can be downloaded at: https://www.mdpi.com/article/10.3390/life15060872/s1.

## Appendix A

**List of recorded taxa from the July studied (J62-86 and J62-64 AST1) wells in the Gulf of Suez, Egypt.**
**Spore**
*Ancyrospora* sp. Figure 8-10
*Aneurospora* sp.
*Apiculatisporites* sp.
*Apiculiretusispora arabiensis* Al-Ghazi. Figure 7-7
*Apiculiretusispora* sp.
*Archaeozonotriletes* cf. *famenensis* Naumova. Figure 8-12
*Archaeozonotriletes* sp.
*Auroraspora solisorta* Hoffmeister Staplin and Malloy. Figure 8-1
*Auroraspora* sp.
*Chelinospora* sp.
*Cirratriradites* sp. Figure 8-11
*Convolutispora* sp.
*Cristatisporites* sp.
*Cymbohilates* sp.
*Cymbosporites* sp.
*Densosporites anulatus* (Loose) Smith and Butterworth. Figure 7-16
*Densosporites* cf. *devonicus* Richardson. Figure 7-15
*Densosporites* sp.
*Diaphanospora* sp.
*Endosporites* sp.
*Geminospora* cf. *lemurata* Figure 8-7
*Geminospora micromanifesta* (Naumova) Owens.
*Geminospora libyensis* Richardson
*Geminospora* sp.
*Grandispora pseudoreticulata* (Menéndez et al.) Ottone
*Grandispora* sp.
*Indotriradites* cf. *explanatus* (Luber) Playford. Figure 8-2
*Hymenozonotriletes* sp.
*Indotriradites* sp.
*Knoxisporites* sp.
*Leiotriletes* sp.
*Microreticulatisporites* sp. Figure 7-13
*Punctatisporites* cf. *irrasus* Hacquebard. Figure 7-8
*Punctatisporites* cf. *solidus* Hacquebard
*Punctatisporites gretensis* Balme and Henelly. Figure 7-1
*Punctatisporites planus* Hacquebard. Figure 7-3
*Punctatisporites* sp. Figure 7-2
*Pustulatisporites* sp. Figure 7-11
*Retusotriletes* sp.
*Retusotriletes* cf. *incohatus* Sullivan. Figure 7-10
*Retusotriletes warringtonii* Richardson and Lister. Figure 7-14
*Rhabdosporites* sp. Figure 8-4
*Samarisporites* sp.
*Samarisporites triangulatus* Allen
*Spelaeotriletes* sp.
*Synorisporites* sp.
*Synorisporites verrucatus* Richardson and Lister
*Vallatisporites* cf. *vallatus* Hacquebard.
*Vallatisporites ciliaris* (Luber) Sullivan
*Vallatisporites pusillites* (Kedo) Dolby and Neves.
*Vallatisporites* sp. Figure 8-5
*Vallatisporites vallatus* Hacquebard.
*Vallatisporites verrucosus* Hacquebard.
*Verrucosisporites nitidus* (Naumova) Playford
*Verrucosisporites* sp. Figure 7-5
*Verrucosisporites verrucosus* (Ibrahim) Ibrahim. Figure 7-12
**Megaspore**
*Lagenoisporites* type. Figure 9-2
**Microphytoplankton**
*Leiosphaerida* sp.
*Lophosphaeridium* sp.
*Schizocystia bicornuta* Jardinè. Figure 9-6.
**Fungi**
Filamentous green algae. Figure 9-5
**Non-spore**
Cuticle-like sheet. Figure 9-3.
Cuticle of arthropods
Plant remains. Figure 9-4.
Tracheid
