# Supplementary material for: Lowermost Carboniferous (Tournaisian) Miospore Assemblages from the July Field, Gulf of Suez, Egypt: Biostratigraphic and Palaeoenvironmental Implications
Source: Life (Basel). 2025 May 28;15(6):872. doi: 10.3390/life15060872 (PMC12194005; doi:10.3390/life15060872)
Supplement: Supplementary file 1 [file life-15-00872-s001.zip › life-3567329-supplementary.pdf]

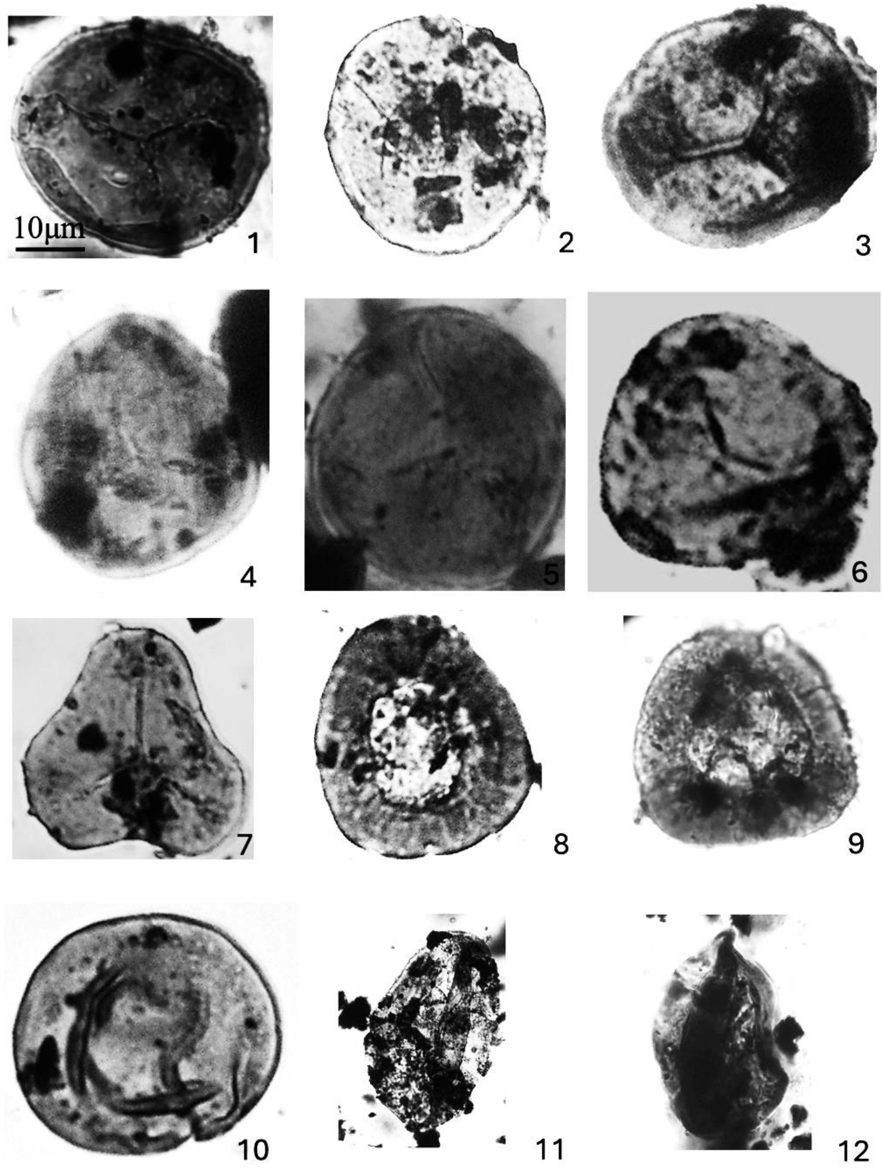

Figure S1. 1-2. *Punctatisporites* sp. 3. *Punctatisporites* cf. *solidus*, J2-09-2: EF. Y2. 4-5. *Retusotriletes* sp. 6. *Leiotriletes* sp./ *L. adnatoides*, J2-09-B-9. 7. *Waltzispora* sp. 8-9. *Densosporites* sp.11-12. *Leiospherids* sp.

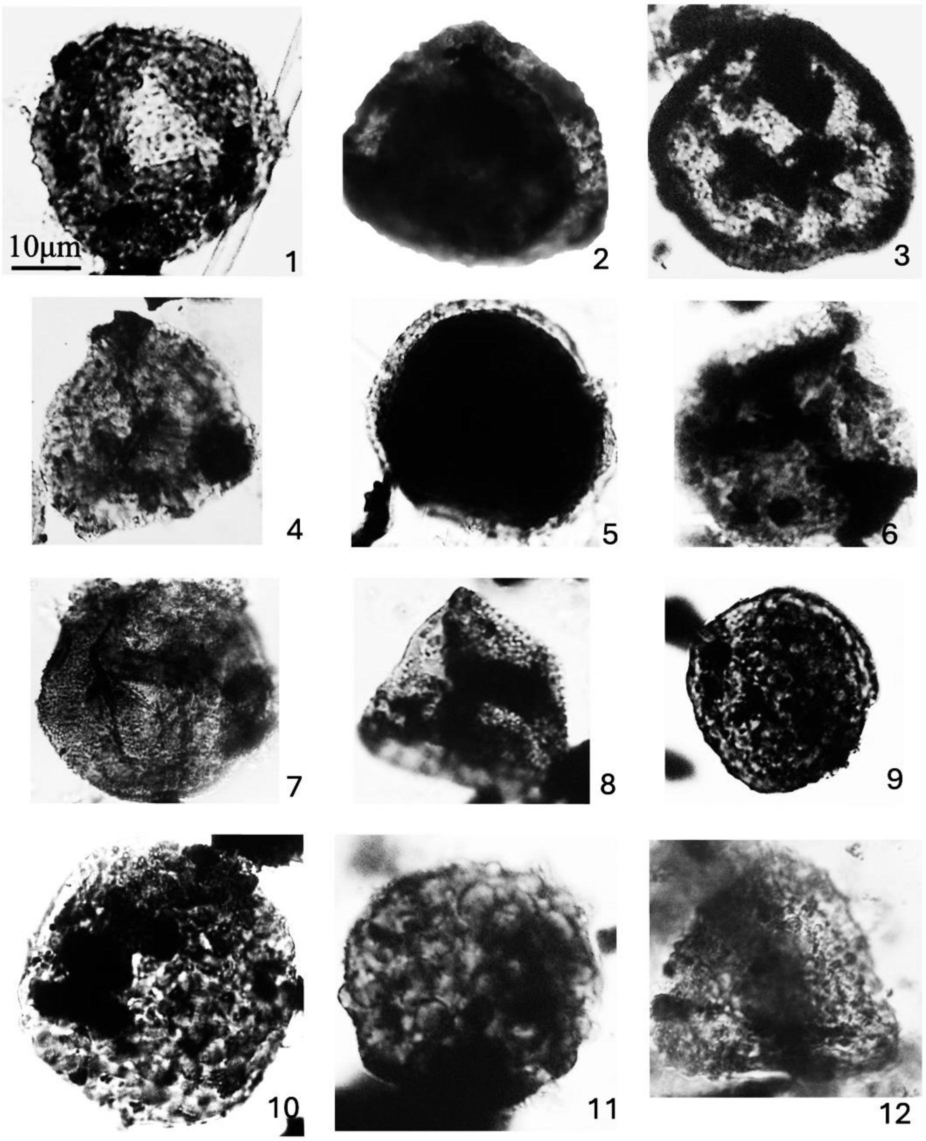

Figure S2. 1. *Verrucosisporites* sp.; J2-09-A8. 2. *Auroraspora* sp. 3. *Archaeozonotriletes famenensis*; J2-09-AZ-5. 4. *Diaphanospora* sp. 5. *Acinosporites lindlarensis*?; J2-03-1: EF. W33. 6. *Samarisporites* sp. 7. *Verrucosisporites* sp.; J2-03-1. 8. *Dibolisporites* sp.; J2-09-D2. 9. *Convolutispora* sp. 10. *Verrucosisporites* sp. 11. *Convolutispora* sp. J2-09-A1: M38. 12. *Synorisporites* sp.; J2-03-1.

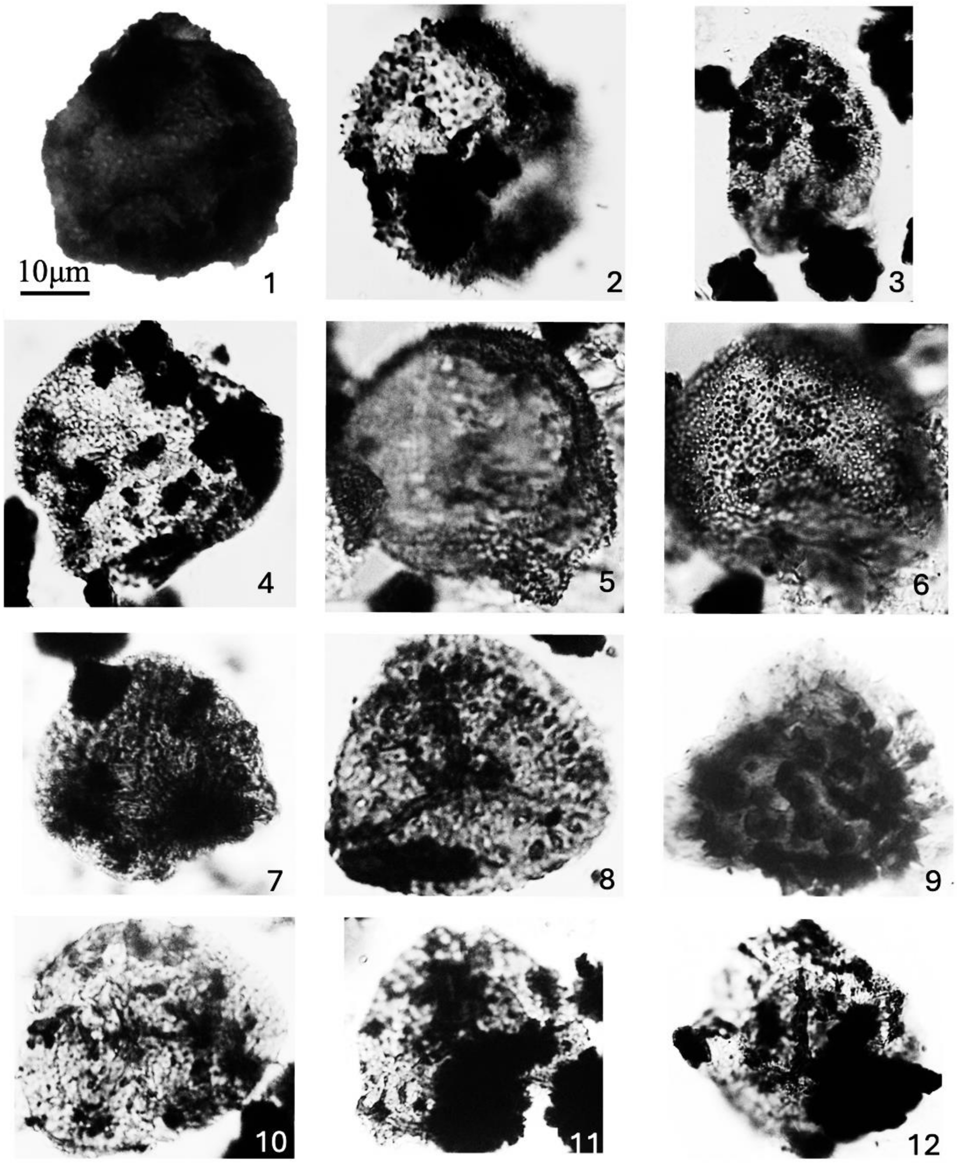

Figure S3. 1. *Chelinospora* sp. 2. *Acinosporites* sp./ *Dibolisporites varius*, J2-09-D1-1. 3, 4. *Apiculiretusispora* sp. 5-6. *Apiculiretusispora* sp.; J2-03-1. 7. *Vallatisporites agadesi*, J1-012-Z1. 8. *Vallatisporites vallatus*, J2-09-B-6: EF. X38. 9. *Vallatisporites pusilites*, J2-03-X1. 10. *Grandispora sola*. 11.?*Grandispora* sp.; J2-09-D1.12. *Grandispora* sp.; J2-09-B11.

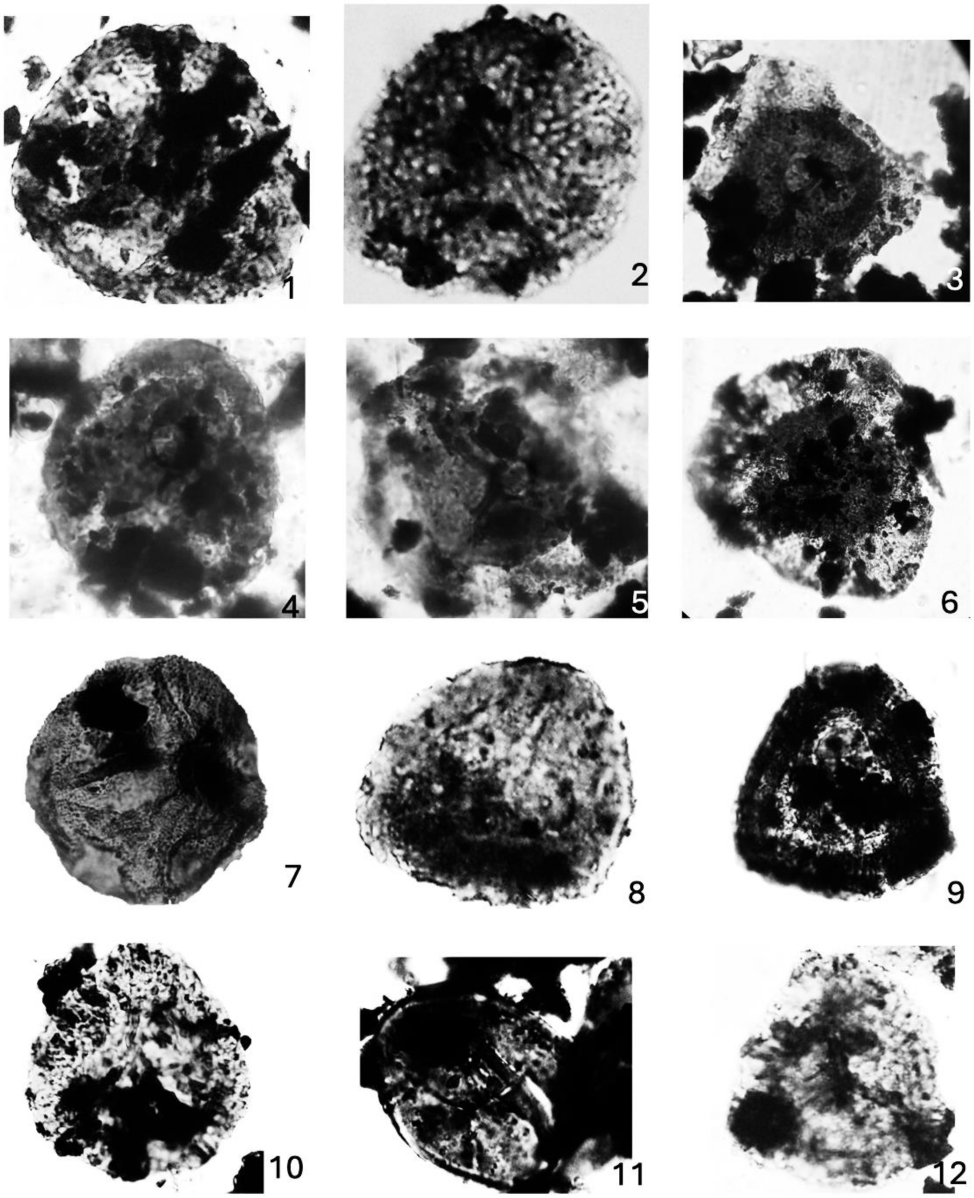

Figure S4. 1. *Rhabdosporites* sp.; J2-09-B8: EF. E55. 2. ?*Aneurospora conica* . 3. *Auroraspora* sp. 4. *Auroraspora* sp. 5. *Auroraspora* sp. 6. *Auroraspora solisorta*; J1-08-6 and J1-08-A1: EF. C20/2. 7. *Lophosphaeridium* sp.; J2-03-1. 8. *Densosporites spitsbergensis*; J2-023-4: EF. E1. 9. *Densosporites* sp. 10. *Archaeozonotriletes* sp.; J2-09-B1. 11. *Acinosporites* sp./ *Dibolisporites varius*; J2-09-D1-1. 12. *Diaphanospora* sp.

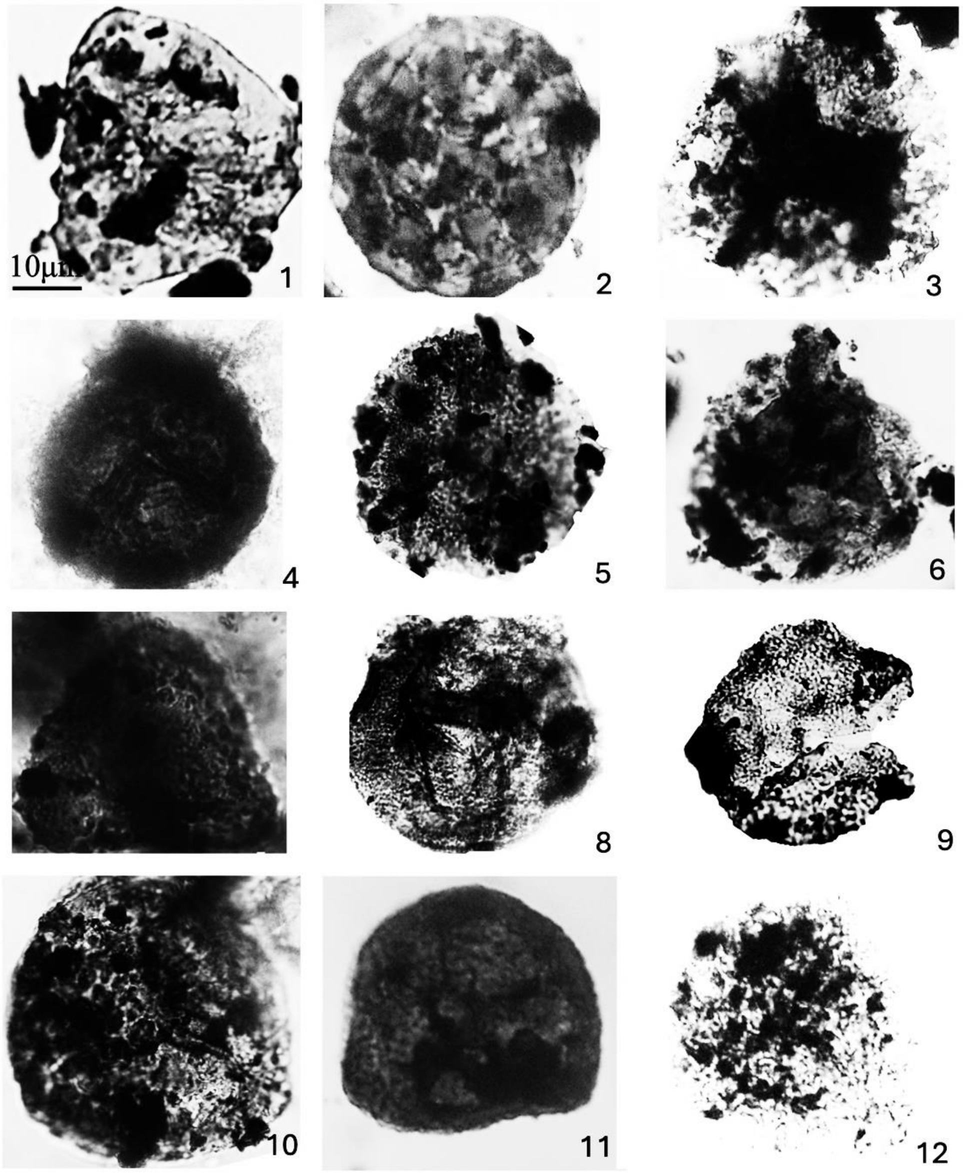

Figure S5. 1. *Anapiculatisporites* sp. 2. *Convolutispora* sp. 3. *Cristatisporites* sp. 4. *Acinosporites lindlarensis*. 5. *Cirratriradites* sp. 6. *Samarisporites* sp. 7. *Synorisporites* sp.; J2-03-1. 8. *Verrucosisporites* sp.; J2-03-1. 9. *Apiculiretusispora* sp.; J1-08-D: EF. Y39/3. 10. *Hymenozonotriletes* sp. 11. *Cymbosporites*/*Synorisporites* complex; J2-09-B-6.12. *Indotriradites* sp.

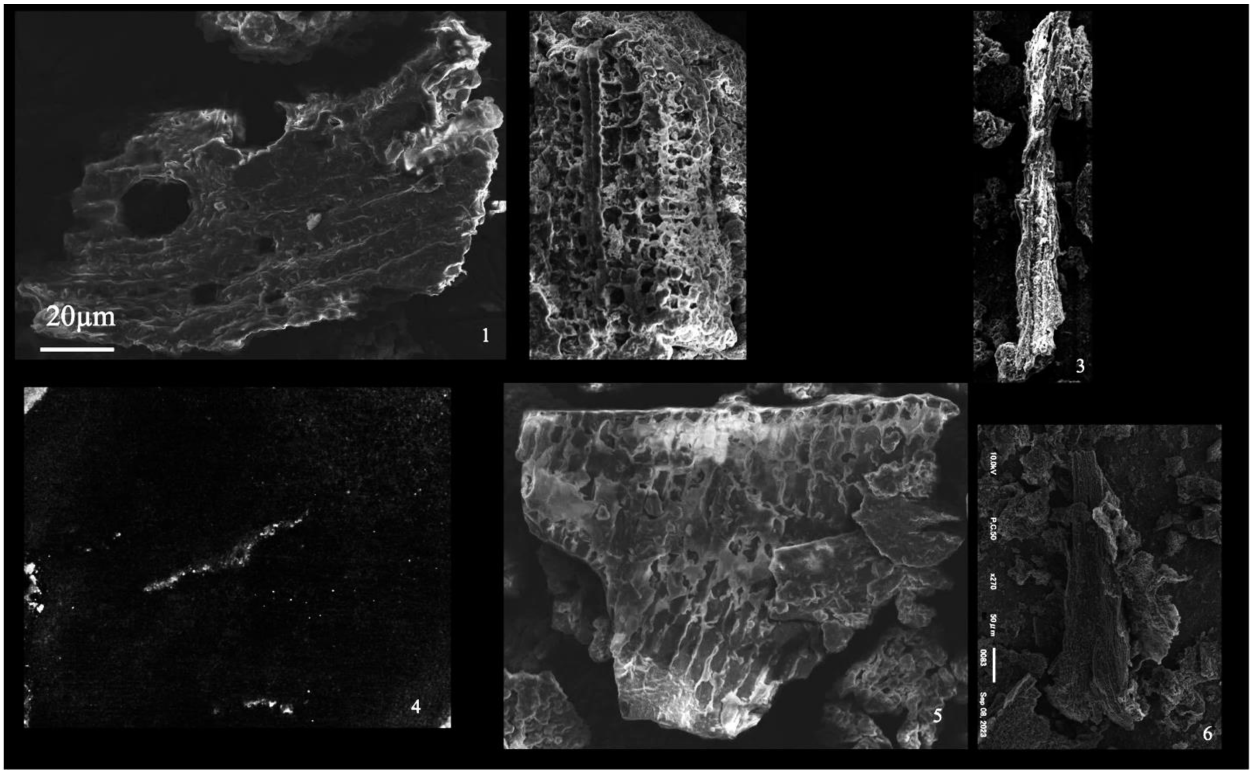

Figure S6. 1. Cuticles. 2. SEM of tracheid. 3 and 6. Tube. 4. Petrographic thin section shows Conodonts. 5. Cuticle-like sheet.

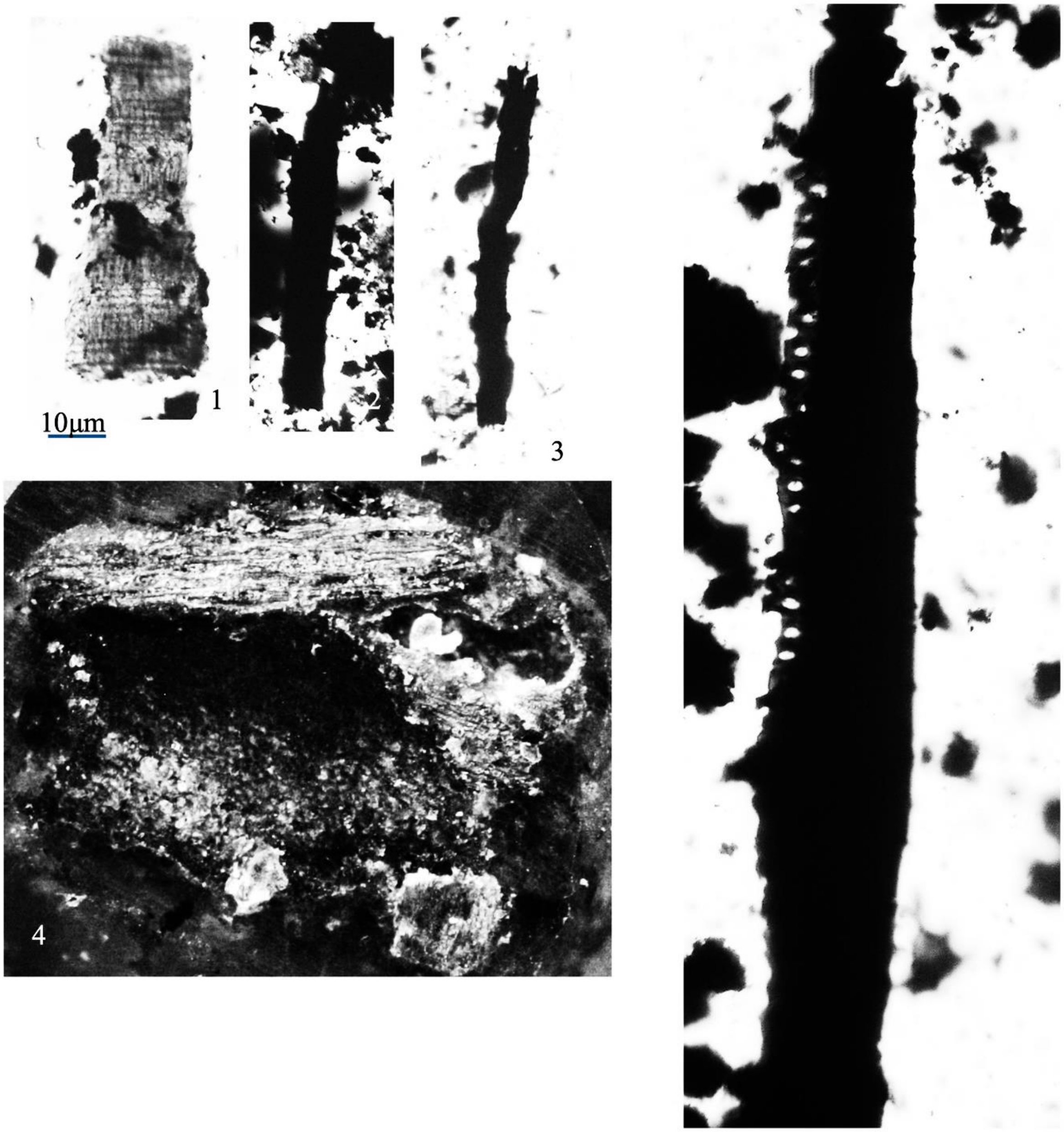

Figure S7. 1. Fragment of arthropod. 2-3. Laevigate tube. 4. The petrographic thin section shows wood. 5. ? Tracheids.

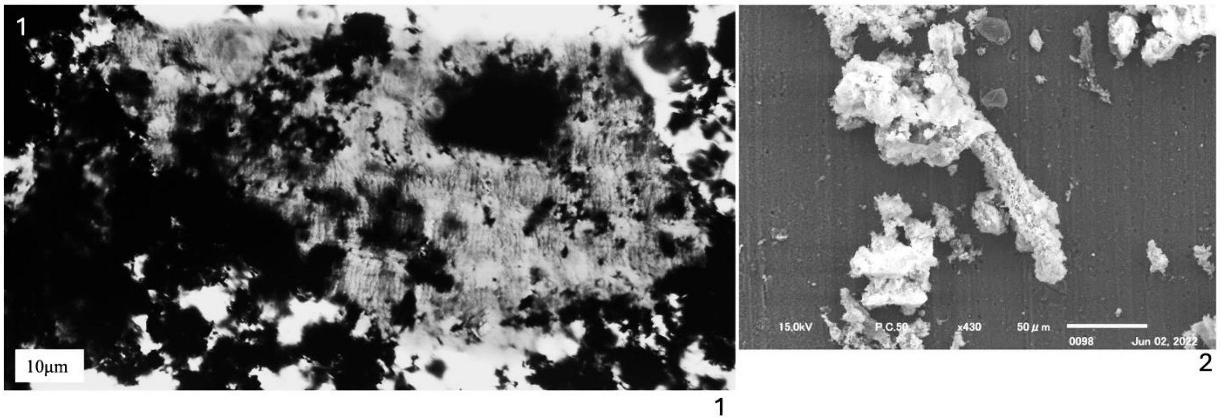

Figure S8. 1. Unknown cuticles. 2-4. Unknown affinity.

A list of recorded taxa in the study of July Field is mentioned in the supplementary file.

Punctatisporites sp.

Punctatisporites cf. *solidus*

Retusotriletes sp.

Leiotriletes sp./ *L. adnatoides*

Waltzispora sp.

Densosporites sp.

Leiospherids sp.

Verrucosisporites sp.

Auroraspora sp.

Archaeozonotriletes famenensis

Diaphanospora sp.

Acinosporites lindlarensis?

Samarisporites sp.

Dibolisporites sp.

Convolutispora sp.

Synorisporites sp.

Chelinospora sp.

Acinosporites sp./ *Dibolisporites varius*

Apiculiretusispora sp.

Vallatisporites agadesi

Vallatisporites vallatus

Vallatisporites pusilites

Grandispora sola

Grandispora sp.

Rhabdosporites sp.

Aneurospora Konica

Auroraspora solisorta

Lophosphaeridium sp.

Densosporites spitsbergensis

Archaeozonotriletes sp.

Anapiculatisporites sp.

Cristatisporites sp.

Acinosporites lindlarensis

Hymenozonotriletes sp.

Cymbosporites/ Synorisporites complex

Indotriradites sp.

Non-Spore Species

Cuticles

SEM of tracheid

Tube

Cuticle-like sheet

Fragment of arthropod

Laevigate tube

? Tracheids

Unknown cuticles

Unknown affinity
